# “Am I normal?” The Wishes of Patients With Lymphoma to Compare Their Patient-Reported Outcomes With Those of Their Peers

**DOI:** 10.2196/jmir.7079

**Published:** 2017-08-15

**Authors:** Simone Oerlemans, Lindy P Arts, Nicole J Horevoorts, Lonneke V van de Poll-Franse

**Affiliations:** ^1^ Department of Research Netherlands Comprehensive Cancer Organisation Utrecht Netherlands; ^2^ Centre of Research on Psychology in Somatic Diseases (CoRPS) Tilburg University Tilburg Netherlands; ^3^ Division of Psychosocial Research and Epidemiology Netherlands Cancer Institute Amsterdam Netherlands

**Keywords:** lymphoma, health-related quality of life, personalized feedback, self-care, access to information, population-based research

## Abstract

**Background:**

Providing feedback to patients on their patient-reported outcomes (PROs) can help patients in monitoring their functioning and symptoms and may help empower them.

**Objective:**

The objective of this study was to investigate whether patients with lymphoma wished to receive PRO feedback, including the option to compare their scores with those of their peers, and how this feedback was evaluated.

**Methods:**

We invited 64 patients participating in a lymphoma cohort who were eligible for a follow-up questionnaire and gave them the option to receive PRO feedback. Patients completed questions about health-related quality of life (HRQoL) and symptoms. PRO feedback was provided via bar charts.

**Results:**

Of the 64 invited patients, 45 participated (response rate 70%) and 36 of those (80%) wished to receive PRO feedback. The vast majority (34/36, 94%) compared their scores with those of a lymphoma reference cohort, and 64% (23/36) compared their score with those of a normative population without cancer. All patients wished to receive feedback on their HRQoL, and 29 (81%) to 33 (92%) wanted feedback on their functioning, fatigue, neuropathy, anxiety, and depressive symptoms. Of the 36 participants wishing to receive PRO feedback, 35 (97%) viewed it as being useful, with reassurance and knowledge about their own functioning in relation to what is “normal” being the most frequently mentioned reasons.

**Conclusions:**

A high number of patients with lymphoma wished to receive PRO feedback. Patients reported the comparison of their scores versus a lymphoma reference cohort as most valuable. Further research should investigate whether PRO feedback could increase empowerment and possibly improve HRQoL.

## Introduction

Patients with lymphoma are at risk of experiencing adverse physical and psychosocial effects of their cancer and its treatment, such as fatigue, cognitive problems, anxiety, and depression [1-4]. Management of these symptoms is often complex, and patients do not always know if their symptoms are common and are caused by their disease or treatment [5].

Patient-reported outcomes (PROs) provide information about the subjective well-being of patients [6]. PROs are standardized questionnaires that are completed by patients and measure a broad range of health-related constructs, including symptom assessment, and evaluation of function and health-related quality of life (HRQoL) [6,7]. Regular screening of physical and psychosocial symptoms by use of PROs could increase awareness and recognition of symptoms and can contribute to managing them [7-11]. PROs are furthermore useful in identifying issues that are most bothersome to patients [12] and can enable patients and their health professionals to jointly identify goals and priorities for future health and health care [13].

The use of PROs in clinical practice has increased in the past years [14]. Studies have shown that feedback from PROs can lead to improved symptom detection and more dialogue about problems between patients and physicians [7-11,15-19]. However, some studies reported no benefit from PRO feedback in the number of patients referred to psychosocial care or in clinical actions taken [16,18,20,21]. In most of these studies, PRO feedback was provided to a health care provider (eg, a physician or nurse). A limitation of providing feedback to health care providers might be that they may not always see the urgency of a specific problem and forget to discuss it. Some health care providers were found to downgrade or miss symptoms such as fatigue and pain [22-24]. Physicians are furthermore most interested in PRO scores that indicate worsening symptoms, whereas patients prefer to see both worsened and improved scores [25]. The provision of PRO feedback to patients themselves might therefore be another and maybe better solution. Patients can then monitor all symptoms and initiate discussion on symptoms that bother them the most. Patients are moreover best placed to interpret their own subjective PROs within the complex context of their experience [26]. Patients also report that the inclusion of PROs in their clinical follow-up made them feel more in control of their care [27].

Comparison of a patient’s outcomes with those of patients with the same age and sex may help to reassure that patient that what he or she is experiencing is “normal” or may empower the patient to take action. The aim of this study was therefore to investigate whether patients with lymphoma wished to receive PRO feedback including the option to compare their scores with those of their peers. We furthermore investigated how patients evaluated the PRO feedback. We hypothesized that around two-thirds of patients would express a wish to receive feedback, as research shows that about 62% of patients with lymphoma want to be fully informed about their illness [28].

## Methods

### Participants and Setting

This study was part of the Patient Reported Outcomes Following Initial Treatment and Long Term Evaluation of Survivorship (PROFILES) lymphoma registry [29]. This is a longitudinal population-based observational study whereby patients with Hodgkin lymphoma and non-Hodgkin lymphoma as diagnosed by the Netherlands Cancer Registry in 9 hospitals in the Netherlands complete questionnaires either on paper or online for research purposes. The first patients were included in 2009, and each year patients with a new diagnosis between 9 months and 1.5 year after diagnosis are invited for questionnaire completion. Patients with a diagnosis within less than 3 years are invited to complete a questionnaire every 6 months and patients with a diagnosis more than 3 years ago are invited to complete a questionnaire once a year. In January 2016, we invited patients with a diagnosis made less than 3 years previously and who were eligible for a follow-up questionnaire to participate in this study. Patients who participated online were given an option to receive PRO feedback. We obtained ethical approval for this study from a certified medical ethics committee (METC Brabant, the Netherlands; reference number: NL54096.028.15/P1533).

### Questionnaire

The questionnaire completed by patients consisted of the following.

We used the Dutch validated version of the European Organization for Research and Treatment of Cancer Quality of Life Questionnaire Core 30 (EORTC QLQ-C30) to assess HRQoL [30]. We added the symptom tingling in hands or feet, as it appeared from the literature and focus groups that this might be a prevalent symptom among patients with lymphoma. Answer categories range from 0 (not at all) to 4 (very much). After linear transformation, all scales and single item measures range from 0 to 100 [30].

We used the Hospital Anxiety and Depression Scale (HADS) [31] to measure anxiety and depressive symptoms in separate subscales of 7 items each. Answers range from 0 to 3, and we calculated scores by adding the items, with a higher score meaning more anxiety or depressive symptoms [31].

We also assessed patients’ marital status, educational level, and comorbidity in the questionnaire and categorized comorbidity at the time of the survey according to the adapted Self-Administered Comorbidity Questionnaire [32]. We obtained clinical characteristics (ie, sex, age, type of lymphoma, date of diagnosis, stage at diagnosis, and primary treatment) from the Netherlands Cancer Registry.

### Procedure

Eligible patients received a letter or email with an invitation to complete the questionnaire. Patients were informed that when they completed the questionnaire online they would have the possibility to receive PRO feedback. After completing the online questionnaire, patients received the following question: “Would you like to receive feedback on your answers to the questionnaire?” If patients answered yes, we asked them on what topics they would like to receive feedback. They could choose from general quality of life, physical functioning, emotional functioning, cognitive functioning, social functioning, fatigue (based on their scores on the EORTC QLQ-C30), tingling hands or feet (based on their score on the question with respect to tingling hands or feet), anxiety or worries, and depressive symptoms (based on their scores on the HADS), or all topics. Subsequently, patients were asked whether they only wanted to see their own scores, and whether they would like to compare their scores with those of other patients with lymphoma or with those of people without cancer, or both. After that, the feedback was generated automatically by computer and was directly shown on the patients’ screens. If patients indicated that they did not wish to receive feedback, the feedback was not generated. Patients who viewed their PRO feedback received evaluation questions afterward.

### Patient-Reported Outcome Feedback

We based the content and layout of the PRO feedback on examples in the literature [33,34] and on lymphoma patients’ preferences reported in an earlier survey on how to provide PRO feedback. In this survey, we presented respondents with 2 examples of PRO feedback: in a bar chart and in a line chart. Respondents had a slight preference for the bar chart. Several examples of PRO feedback presented as bar charts were subsequently evaluated by 12 persons (mean age 55 years; 8/12, 67% female; 5/12, 42% low level of educational attainment). We asked them which colorway they preferred: traffic light colors, pastel colors, or PROFILES house-style colors. Here respondents preferred traffic light colors. Patients furthermore preferred a dotted line over a solid line to indicate “your score” in the bar chart. In this study, we therefore provided the PRO feedback via bar charts in traffic light colors with a dotted line to indicate a patient’s score.

**Figure 1:**
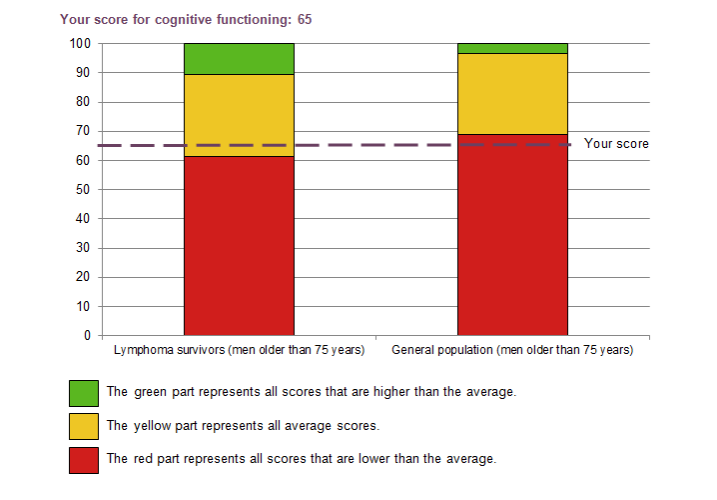
The example of cognitive functioning as part of patient-reported outcome feedback provided to participants.

If patients wanted to view their own scores, a single bar chart was shown for each PRO feedback topic. If patients had indicated that they wanted to compare their scores with those of a lymphoma reference cohort or a normative population without cancer, both of the same sex and age, either 1 or 2 traffic light-colored bar charts were shown (see Figure 1 for an example). Age was grouped into categories of 10-15 years, ranging from 18-30 years to older than 75 years. The colors of the bar charts were related to clinically relevant mean differences of the evidence-based guidelines of the EORTC QLQ-C30 [35]. A score that differed by less than the minimal medium clinically relevant difference from the mean score was considered average (amber). A score that differed as much as or more than the minimal medium clinically relevant difference from the mean score was considered above average (green) or below average (red). We interpreted anxiety and depressive symptoms according to the published scoring algorithm with 0-7 indicating no or mild symptoms (green), 8-10 indicating moderate symptoms (amber), and ≥11 indicating severe symptoms (red) [31]. We added a detailed description of the meaning of the colors (traffic light model) and how to interpret the scores to assist patients in understanding the graphs (Textbox 1 shows cognitive functioning as an example). Patients with a symptom score in the red part of the bar chart were advised to contact their general practitioner.

**Description of cognitive functioning (concentration and memory) as an example for interpreting the bar charts.**

Cognitive functioning is a component of quality of life. Cognitive functioning, for example, refers to the extent to which one can concentrate or can remember things.

On the cognitive function component, you can score between 0 and 100. The higher the score, and the closer the score is to 100, the higher you will experience your quality of life in this part. Your score is shown in the graph by the purple line.

Your score in comparison with *other lymphoma survivors:*

- Your score falls in the yellow part. This indicates that your score is similar to that of other people with lymphoma with your age and sex.

Your score in comparison with *the general population:*

- Your score falls in the red part. This indicates that your score is lower than the average score of people from the general population with your age and sex.

People with lymphoma score generally lower on cognitive functioning than the general population. Memory and concentration problems are common among people with cancer. Some also experience difficulty working under time pressure or doing different things at the same time. Others must make a greater mental effort to reach the same results as when they were living without cancer. [36]

### Lymphoma Reference Cohort and Normative Population

We based the mean scores of the lymphoma reference cohort on data from our previous population-based study on HRQoL among 856 patients with lymphoma [37]. We extracted the mean scores of an age- and sex-matched normative population of 1859 individuals without cancer from a reference cohort from the general Dutch population (CentER panel) [38].

### Evaluation Questions

The evaluation questions consisted of 5 open questions with respect to the usefulness, accessibility, clarity, and missing features of the feedback. Patients were furthermore asked whether they would have liked to see different features in the PRO feedback. Based on the average scores on HRQoL and anxiety and depressive symptoms, we evaluated whether both patients with and patients without symptoms wanted to receive PRO feedback.

### Statistical Analysis

Analyses were performed using SAS version 9.1 (SAS Institute Inc). *P*<.05 were considered statistically significant. We determined clinically relevant differences using the evidence-based guidelines of the EORTC QLQ-C30 [35].

We used Fischer exact tests or *t* tests to compare differences in sociodemographic and clinical characteristics between respondents and nonrespondents and between patients who wished and those who did not wish to receive PRO feedback.

To evaluate whether scores were on average comparable with those of a lymphoma reference cohort, we compared patients’ mean EORTC QLQ-C30 and HADS scores with mean scores of a lymphoma reference group using analysis of covariance with age and sex as covariates. We also compared patients’ mean scores, in the same way, with those of a normative population. The numbers of patients scoring in the red, amber, or green part were computed to evaluate whether both patients with and patients without symptoms wished to receive PRO feedback.

## Results

### Participants

Of the 64 patients who were invited, 45 participated (response rate 70%). Their mean age was 60.7 years and 58% (n=26) were male. Mean time since diagnosis was 2.8 years, and 82% (n=37) had a diagnosis of non-Hodgkin lymphoma. Most patients underwent systemic therapy or radiotherapy, or both. Sociodemographic and clinical characteristics did not statistically differ between respondents and nonrespondents (Table 1).

### Evaluation of Patient-Reported Outcome Feedback

A total of 36 (80%) participants wished to receive PRO feedback, with similar percentages for males and females (21/26, 81% vs 15/19, 79%; *P*=.29) and for patients under and above 65 years of age (20/26, 77% vs 16/19, 84%; *P*=.25). Patients who wished to receive PRO feedback had scores on overall HRQoL (*P*=.14) and anxiety (*P*=.47) and depressive symptoms (*P*=.25) similar to those of patients who did not wish to receive feedback.

The vast majority (34/36, 94%) compared their scores with those of the lymphoma reference cohort and 64% (23/36) compared their scores with those of the normative population without cancer, whereas 6% (2/36) viewed only their own scores.

All patients viewed the PRO feedback on their overall HRQoL, and 81% to 92% viewed feedback on their physical, emotional, social, and cognitive functioning, fatigue, tingling in hands or feet, anxiety, and depressive symptoms (Table 2).

**Table 1:** Sociodemographic and clinical characteristics of respondents and nonrespondents.

Characteristics		Respondents (n=45)	Nonrespondents (n=19)	*P* value, respondents vs nonrespondents
**Sex, n (%)**				.27
	Male	26 (58)	14 (74)	
	Female	19 (42)	5 (26)	
**Age in years, mean (SD)**		60.7 (13.6)	63.8 (14.7)	.28
	<65, n (%)	26 (58)	8 (42)	
	≥65, n (%)	19 (42)	11 (58)	
**Marital status, n (%)**				
	Partner	34 (76)	N/A^a^	
	No partner	11 (24)	N/A	
**Educational level attained, n (%)**				
	Secondary	8 (18)	N/A	
	Intermediate vocational	17 (38)	N/A	
	High vocational or university	20 (44)	N/A	
**Type of lymphoma, n (%)**				.26
	Hodgkin	8 (18)	1 (5)	
	Non-Hodgkin	37 (82)	18 (95)	
Years since diagnosis at time of questionnaire completion, mean (SD)		2.8 (0.8)	2.6 (0.7)	.84
**Cancer stage at diagnosis, n (%)**				.50
	I	8 (22)	4 (29)	
	II	10 (28)	2 (14)	
	III	5 (14)	4 (29)	
	IV	13 (36)	4 (29)	
**Primary treatment, n (%)**				.11
	Radiotherapy only	2 (4)	1 (5)	
	Systemic therapy (eg, chemotherapy, immunotherapy)	19 (42)	8 (42)	
	Systemic therapy plus radiotherapy	13 (29)	1 (5)	
	Active surveillance	11 (25)	9 (47)	
Self-reported comorbidities, mean (SD)		1.3 (1.3)	N/A	
**Most frequently reported self-reported comorbidities, n (%)**				
	Arthritis	10 (22)	N/A	
	Heart problems	8 (18)	N/A	
	High blood pressure	8 (18)	N/A	

^a^N/A: not available.

**Table 2:** Overview of patient-reported outcome feedback topics with number and percentage of interested patients.

Topic		n	%
**EORTC QLQ-C30^a^**			
	General HRQoL^b^	36	100
	Physical functioning	33	92
	Emotional functioning	32	89
	Social functioning	33	92
	Cognitive functioning	31	86
	Fatigue	31	86
	Neuropathy	29	81
**HADS^c^**			
	Anxiety	30	83
	Depressive symptoms	30	83

^a^EORTC QLQ-C30: European Organization for Research and Treatment of Cancer Quality of Life Questionnaire Core 30.

^b^HRQoL: health-related quality of life.

^c^HADS: Hospital Anxiety and Depression Scale.

Almost all patients (except 1) viewed the PRO feedback as being useful, with reassurance and knowledge about their own functioning in relation to what is “normal” being the most frequently mentioned reasons. The option to compare their scores with those of a lymphoma reference cohort of the same age and sex was reported as most valuable:

This score shows what I actually did expect of my quality of life. The comparison with other patients with lymphoma feels right. I mean, I do not score that different and that again reassures me.

Female patient with non-Hodgkin lymphoma, 69 years old

It is interesting to see how I stand compared to other patients with lymphoma and the general population.

Male patient with Hodgkin lymphoma, 22 years old

The PRO feedback clarifies if symptoms are shared by others or not.

Female patient with Hodgkin lymphoma, 37 years old

Some patients reported that the PRO feedback was useful, since it provided new insights for discussion with their physician. No reason was provided by the patient who indicated that the PRO feedback was not useful.

A total of 2 patients reported that the PRO feedback had missing features; 1 patient advised us to provide more information on how to limit symptom burden or improve symptoms; and 1 patient suggested that it would be good to advise patients to go to their general practitioner when experiencing problems:

Not everyone has good and regular contact with their doctors, so it would be helpful to advise a patient to contact a doctor when he or she reports problems.

Female patient with non-Hodgkin lymphoma, 54 years old

The comment regarding contacting a general practitioner was already covered for the symptoms in the current PRO feedback for patients scoring in the red part of the bar chart, but not for the functioning scales.

With respect to the clarity of the PRO feedback, 1 patient missed the possibility to go back to his answers in the questionnaire to verify that the PRO feedback was correct, because his score on neuropathy was very low according to the PRO feedback, but not in his experience. Furthermore, 1 patient had trouble understanding the colors of the PRO feedback at first, but after looking for a second time it became clear. With respect to things that should be different, some patients indicated that they wished to save their scores for future comparison purposes and to keep track of their scores:

Is it possible to download my PRO feedback, so I can compare my scores over time and determine potential deterioration?

Male patient with non-Hodgkin lymphoma, 84 years old

### Health-Related Quality of Life, Anxiety, and Depression Scores

Mean scores on HRQoL, anxiety, and depressive symptoms of participating patients in this study were not different from the mean scores of the lymphoma reference cohort (Table 3). Compared with the normative population, patients had on average statistically and clinically relevant lower scores on physical, cognitive, and social functioning and higher scores on fatigue (all *P*<.05).

With respect to patients’ individual scores on HRQoL, 33% (n=15) of patients reported scores that were lower than the average of the lymphoma reference cohort (red part of bar chart) and 31% (n=14) reported scores higher than the average range of scores (green part of bar chart; Table 4). Compared with the normative population, 33% (n=15) of patients reported scores that were lower than the average and 20% (n=9) reported scores higher than the average of the normative population. The percentages were similar for the other scales (data not shown).

**Table 3:** EORTC QLQ-C30^a^ + tingling hands or feet and HADS^b^ mean scores of patients, a lymphoma reference cohort, and a normative population, and clinically important differences between these groups.

Measure		Patients (n=45)	Lymphoma reference cohort (n=876)	Normative population (n=1852)	Patients vs lymphoma cohort		Patients vs normative population	
					*P* value^c^	Clinical relevance	*P* value^c^	Clinical relevance
**EORTC QLQ-C30, mean (SD)**								
	Physical functioning	83.1 (20)	79.4 (21)	90.5 (15)	.21	No	<.001	Small
	Emotional functioning	82.2 (21)	82.8 (21)	87.9 (17)	.86	No	.02	Trivial
	Cognitive functioning	80.4 (22)	82.4 (23)	92.5 (14)	.57	No	<.001	Medium
	Social functioning	85.9 (25)	84.4 (24)	93.6 (16)	.68	No	.002	Small
	Global health status/QoL^d^	73.3 (20)	74.0 (20)	77.6 (17)	.82	No	.10	Small
	Fatigue	24.7 (23)	28.9 (27)	17.0 (20)	.30	No	.01	Small
	Tingling hands or feet	18.5 (28)	17.0 (29)	N/A^e^	.73	No	N/A	N/A
**HADS, mean (SD)**								
	Anxiety	4.0 (3.8)	4.4 (3.8)	3.6 (3.2)	.51	No	.34	No
	Depressive symptoms	3.9 (3.8)	4.7 (3.8)	3.6 (3.2)	.17	No	.54	No

^a^EORTC QLQ-C30: European Organization for Research and Treatment of Cancer Quality of Life Questionnaire Core 30.

^b^HADS: Hospital Anxiety and Depression Scale.

^c^*P* value is adjusted for age and sex.

^d^QoL: quality of life.

^e^N/A: not available.

**Table 4:** Number and percentages of patients scoring lower, similar to, or higher than the lymphoma reference cohort and normative population on EORTC QLQ-C30^a^ global health status/quality of life.

Relative scores	Compared with lymphoma reference cohort	Compared with normative population
Lower than average (red)	15 (33)	15 (33)
Average (amber)	16 (36)	21 (47)
Higher than average (green)	14 (31)	9 (20)

^a^EORTC QLQ-C30: European Organization for Research and Treatment of Cancer Quality of Life Questionnaire Core 30.

## Discussion

### Principal Findings

Of the participating patients with lymphoma, 80% wished to receive PRO feedback, which was higher than the two-thirds of patients that we hypothesized. A similar percentage of men and women and patients younger and older than 65 years wished to receive PRO feedback. They reported the comparison of their scores with those of a lymphoma reference cohort as being very valuable, since it provided information about their functioning in relation to what is “normal.”

An advantage of providing PRO feedback to patients themselves is that patients can monitor their symptoms at any specific point in time. Patients are furthermore provided with information that they can use to actively engage with their physician when discussing symptoms [26,27]. However, not all patients will be self-assertive enough to bring up their problems and, in that case, providing feedback to both patients and physicians, as is done in some studies [16-18], might be more effective for discussing problems and taking action with respect to referral to other health care professionals.

Almost all patients indicated that the PRO feedback was useful and reassuring. Even when patients had scores that were below average, they still viewed PRO feedback as useful. The latter pleads for providing PRO feedback as a standard option in care. However, before PRO feedback is provided, patients need to be asked for their preference, as still 20% indicated that they did not want to receive PRO feedback. This is the case for information provision in general, as patients fare psychologically, behaviorally, and physiologically better when the information they receive about their medical condition is tailored to their coping styles, whereby those with a monitoring style tend to do better when given more information, and those with a blunting style do better with less information [39].

Since the feedback was generated automatically after patients completed the questionnaire, implementation in our PROFILES registry is relatively simple. In addition, providing PRO feedback is valuable for other research that is performed with online questionnaires, as well as for patients with other medical conditions in terms of empowering patients and monitoring their functioning and symptoms.

In this study, we evaluated PRO feedback in a research setting at a fixed time point, but this kind of PRO feedback could also be of merit for patients at any given point in time outside of a research setting. It can, for example, be used as a tool for keeping track of their scores, which may help patients to feel more in control of their cancer and care [27].

### Limitations

The sample size was relatively small, although we obtained a response rate of 70%. The PRO feedback was accessible only to patients completing the questionnaire online, which limits the generalizability of the results to the total lymphoma population, as patient characteristics are different for patients who participated online versus patients who participated on paper [40].

### Conclusion

Future research should determine whether this kind of feedback could also increase empowerment and possibly improve HRQoL.

## Abbreviations

EORTC QLQ-C30: European Organization for Research and Treatment of Cancer Quality of Life Questionnaire Core 30
HADS: Hospital Anxiety and Depression Scale
HRQoL: health-related quality of life
PRO: patient-reported outcome
PROFILES: Patient Reported Outcomes Following Initial Treatment and Long Term Evaluation of Survivorship
